# Individuals with a previous symptomatic COVID-19 infection have altered heart rate and blood pressure variability during acute exercise

**DOI:** 10.3389/fphys.2023.1052369

**Published:** 2023-02-06

**Authors:** Jillian Chan, Hailey Senior, Jessica Homitz, Niamh Cashin, John J. Guers

**Affiliations:** Department of Biology, Behavioral Neuroscience and Health Sciences, Rider University, Lawrenceville, NJ, United States

**Keywords:** COVID-19, exercise, heart rate variability, blood pressure variability, cardiovascular, autonomic nervous system

## Abstract

**Introduction:** As the number of COVID-19 cases begin to diminish it is important to turn our attention to any long-term issues that may be associated with a prior infection. Cardiovascular defects have been noted following prior SARS-CoV-2 infections. However, less is known about how a previous infection alters the cardiovascular response to exercise. Further, differences may exist during exercise between previously SARS-CoV-2 positive individuals who had symptoms (symptomatic) relative to those who did not have symptoms (asymptomatic). We hypothesized that previously symptomatic (S) COVID-19 recoveries have an altered cardiovascular response to acute exercise relative to both control (CON; never infected), and previously COVID-19 positive asymptomatic (AS) individuals.

**Methods:** Twenty-seven subjects (CON = 9; AS = 9; S = 9) underwent 30 min of submaximal treadmill exercise. During exercise, blood pressure was recorded on the brachial artery every 5 min and 3-lead electrocardiography was measured continuously. Indirect indicators of autonomic nervous system health: heart rate variability and blood pressure variability were measured during each session. Baseline mean arterial pressure (MAP) was taken prior to exercise in seated, standing and supine positions.

**Results:** Blood pressure was similar (*p* > 0.05) amongst all three groups. There were no differences between average heart rate (HR; CON = 104 ± 4 BPM vs AS = 118 ± 6 BPM vs. S = 112 ± 3 BPM), mean arterial pressure (MAP; CON = 108 ± 4 mmHg vs. AS = 105 ± 13 mmHg vs. S = 108 ± 7 mmHg) or oxygen consumption (VO_2_) between groups during a bout of exercise. However, the standard deviation of the inter beat intervals of normal sinus beats, a measure of heart rate variability (HRV) (CON = 138 ± 2.8 m vs. AS = 156 ± 6 m vs. S = 77.7 ± 11 m; *p* < 0.05) and blood pressure variability (BPV; CON = 5.18 ± 1.1 vs. AS = 12.1 ± 0.88 mmHg vs. S = 10.2 ± 10.7 mmHg; *p* < 0.05) were different in our S group. Further, when HRV was assessed in the frequency domain the very low frequency was different during exercise in the S group relative to the other groups.

**Discussion:** Collectively, these data suggest that a previous symptomatic SARS-CoV-2 infection may alter heart rate and blood pressure regulation during exercise.

## Introduction

As the number of COVID-19 cases begin to diminish, it is important to turn our attention to any long-term issues that may be associated with a prior infection. Studies have pointed to potential issues with the heart, lungs, and the nervous system (Lotfi et al., 2020; Wu et al., 2020; Veluswamy et al., 2021). Due to the novelty of the virus long term data and general health outcomes of survivors are lacking. Therefore, we are only beginning to understand the long-term consequences of contracting SARS-CoV2. Our data were collected over a span of 2 years (June 2020—June 2022). Although multiple variants emerged during this time point they unlikely have become more virulent (Sheikh et al., 2022).

Studies point to the fact that cardiac abnormalities and disease, such as myocarditis and myocardial infarction, may be a consequence of contracting the SARS-CoV-2 virus (Goha et al., 2020; Mai et al., 2020) and therefore makes exercise a valuable tool to combat a potential onset of future cardiovascular issues as they relate to COVID-19 (Jimeno-Almazán et al., 2021). The modulation of cardiac output and blood pressure during exercise is critical. If blood pressure regulation is altered as a result of a previous SARS-CoV-2 infection it could result in exercise intolerance and ameliorate the potential to use exercise as a cardioprotective measure. Previous work has demonstrated the existence of post COVID-19 exercise intolerance (Ferreira and Oliveira, 2021). This study found a reduction in peak exercise oxygen consumption (VO_2_) during exercise (Ferreira and Oliveira, 2021). Although the mechanisms, are unclear it appears that there is an earlier onset of the subject’s lactate threshold suggesting a level of aerobic deconditioning (Rinaldo et al., 2021; Skjørten et al., 2021). Lastly, it appears that some subjects lightly hyperventilate when faced with exercise and thus are expelling more carbon dioxide earlier on during exercise (Singh et al., 2022). One particular hypothesis was that metaboreceptors become more sensitive as a result of a prior COVID-19 infection and as a result increase ventilatory drive (Ferreira and Oliveira, 2021).

Heart rate variability (HRV) and blood pressure variability (BPV) are linked to cardiovascular health and can be used to assess autonomic nervous system function. Sympathetic and parasympathetic flow are crucial for the beat to beat adjustments that are needed during exercise. HRV can demonstrate a reduction in the regulatory capacity of the nervous system which is necessary to meet the demands of stressors such as exercise. Therefore, this can be a potential mechanism for the exercise intolerance which is document in COVID-19 positive patients. Based on these past studies we were interested if a prior SARS-CoV-2 infection changes the typical cardiovascular responses to exercise. We hypothesized that previously symptomatic (S) COVID-19 recoveries have an altered cardiovascular response to acute exercise relative to both control (CON; never infected), and previously COVID-19 positive asymptomatic (AS) individuals. We aimed assess differences in exercise blood pressure and heart rate regulation between previously symptomatic and asymptomatic COVID-19 positive individuals relative to those who never contracted the virus.

## Materials and methods

Subject Population: 27 male and female recreationally active who reported meeting the American College of Sports Medicine (ACSM) Guidelines for exercise (150 min a week at moderate intensity) and sedentary Rider University students who reported no exercise volunteered to participate in our study. The experimental protocols and the process for obtaining informed consent conformed with the Declaration of Helsinki and were approved by the Institutional Review Board (IRB) at Rider University. Subjects declared during their informed consent if they were previously: 1) COVID-19 Positive with symptoms (symptomatic; S) or 2) COVID-19 positive without symptoms (asymptomatic; AS) or 3) COVID-19 negative (Control) as per confirmation of *via* a polymerase chain reaction (PCR)-based test. All participants were studied at Rider University in the Human Performance Laboratory.

Gas Analysis: A Parvo Medics True One 2,400 Metabolic Testing System (Parvo Medics, Utah, United States) was used to measure oxygen consumption (VO_2_) *via* indirect calorimetry. Each subject was first weighed to ensure the accuracy of our VO_2_ measurements. Breath by breath analysis of oxygen and carbon dioxide was made continuously starting at 5 min prior to exercise and continuing for the duration of exercise and finishing 10 min following exercise.

Treadmill Exercise Bout: Exercise was performed on a Trackmaster Treadmill (Fullvision, Kansas, United States)**.** Each participant underwent a single bout of moderate intensity treadmill exercise. The subjects were connected to a metabolic testing system and began walking at 2.5 mph with a 3.0% incline for 5 min as a warm-up. Following the warm-up subjects were exercised at six METS followed by a 5-min cool-down. VO_2_, electrocardiography (EKG) were measured throughout the duration of the exercise bout and blood pressure was taken every 5 min.

Electrocardiography (EKG): A three lead EKG (Right Arm, Left Arm and Limb Lead) were measured continuously starting at 5 min prior to the onset of exercise and for up to 10 min following the completion of exercise. EKG was monitored using CardioCard v.635 and Cardiosuite software (Nasiff Associates; NY; United States). A 40 Hz low pass filter and a 60 Hz filter was applied to the recorded EKG tracing.

Blood Pressure: Blood pressure was measured using a Suntech Tango M_2_ blood pressure monitor (Suntech, NC, United States) *via* the brachial artery. Because of previously documented orthostatic intolerance following COVID-19 infection (Dani et al., 2021), blood pressure was also measured in a seated, standing and lying position prior to the onset of exercise. During exercise, blood pressure was measured every 5 min and 10 min following exercise.

### Experimental procedure

Blood pressure was taken prior to exercise in the seated, standing, and supine positions. Following these preliminary blood pressure measurements resting EKG and gas analysis were collected for 5 min before subjects began their exercise session with a 5-min warm-up consisting of walking at 2.5 mph with a 3.0% incline on a treadmill. Following the warm-up each subject underwent 20 min of treadmill exercise. The speed was monitored and adjusted according to ensure each subject could complete a full 20-min bout of exercise. The subjects finished with a 5-min cool down at 1.8 mph speed and a 0% incline. EKG was used to measure heart rate and rhythm. Blood pressure was recorded on the brachial artery every 5 min and electrocardiography and breath by breath VO_2_ was measured continuously.

### Data analysis

***Heart Rate Variability (HRV):*** R to R intervals were collected using EKG at a frequency of 250 Hz. Recordings were divided into 5 min intervals throughout the experiment for analysis. The recordings were analyzed using Cardiosuite Software. HRV was analyzed in the time-domain using the standard deviation of the inter beat intervals of normal sinus beats (SDNN), which has been used in previous studies (Shaffer and Ginsberg, 2017). HRV was also analyzed in the frequency domain and the following components were derived from the signal: high frequency (Hf) (0.15–4.0 Hz), low frequency (LF) (0.04–0.15 Hz) and very low frequency (VLF) (0.0033–0.04 Hz).

***Tests of Stationary -*** Each 5-min HRV interval, which occurred during exercise was assessed for violations of stationary. We conducted a standard Dickey-Fuller test in Microsoft Excel (2019) to explore differences in the mean and variance of each subset of HRV relative to the mean and variance of the entire signal. The Null Hypothesis was that the if failed to be rejected, the data was non-stationary.

***Blood Pressure Variability (BPV):*** Brachial artery BPV was computed as the average of the absolute differences between consecutive mean arterial pressure (MAP) measurements.

***Statistical Analysis:*** Figures were created in GraphPad Prism software Version 5.03 (San Diego, CA). Statistical analysis was also run in GraphPad Prism. A two-way mixed model repeated measures ANOVA with a Bonferroni post-hoc test evaluation will be was used to evaluate significance between three groups (Control, COVID-19 symptomatic (S) and COVID-19 asymptomatic (AS) in two conditions (before and during exercise) for heart rate, blood pressure and oxygen consumption. *P* values less than 0.05 will be considered significant and all data is presented as the mean ± standard error (SE).

## Results

**FIGURE 1:**
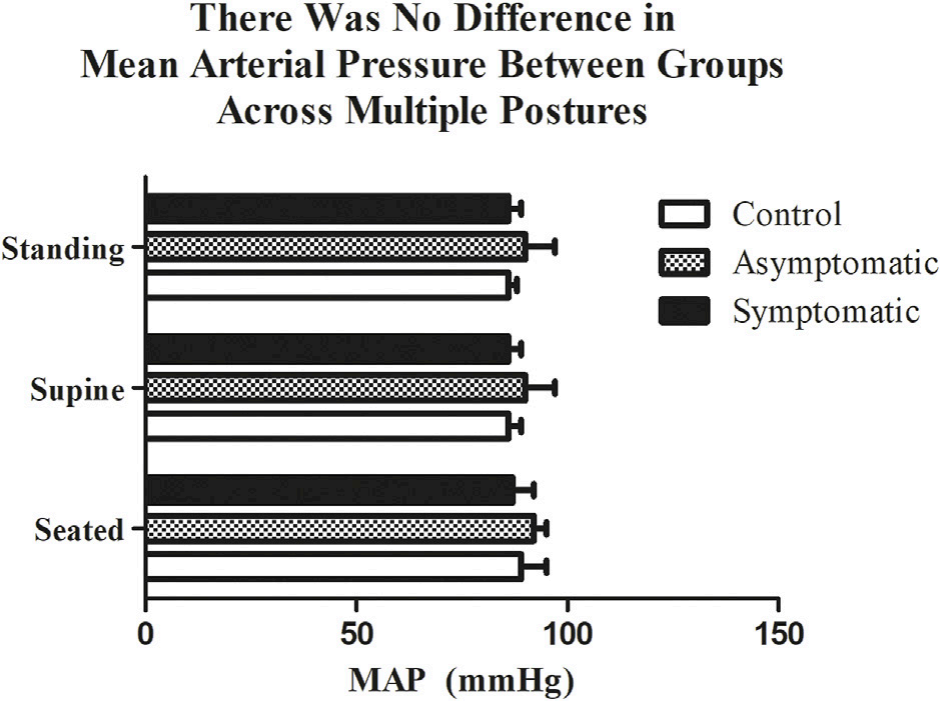
There was no difference in mean arterial pressure (MAP) between groups while standing, seated, or lying. *n* = 9/group. Data analyzed by a Two-Way Repeated Measures ANOVA with a Bonferroni post-hoc. Data are presented as mean ± SE; MAP = mean arterial pressure. Significance was set to *p* < 0.05.

We collected data on 27 subjects (Table 1.) Considering that orthostatic intolerance was noticed following a SARS-CoV-2 infection (Dani et al., 2021), we first measured blood pressure in three different postures (Figure 1). We found that resting MAP was not different between our groups in respect to each posture: seated (CON = 89 ± 6 mmHg; AS = 92 ± 3 mmHg; S = 87 ± 5 mmHg), standing (CON = 86 ± 2 mmHg; AS = 90 ± 7 mmHg; S = 86 ± 3 mmHg) and supine (CON = 86 ± 3 mmHg; AS = 90 ± 6 mmHg; S = 86 ± 4 mmHg).

**TABLE 1:** Demographic data for the participants of this study.

Variable	Female participants (*n* = 18)	Male participants (n = 9)
Age (year)	21.1 ± 0.7	20.6
Height (cm)	165.1 ± 4.9	175.3 ± 6.1
Body Mass (kg)	68.3 ± 5.7	82.8 ± 6.7
Weekly Exercise Total (mins)	123 ± 14.2	109 ± 19.1

**FIGURE 2:**
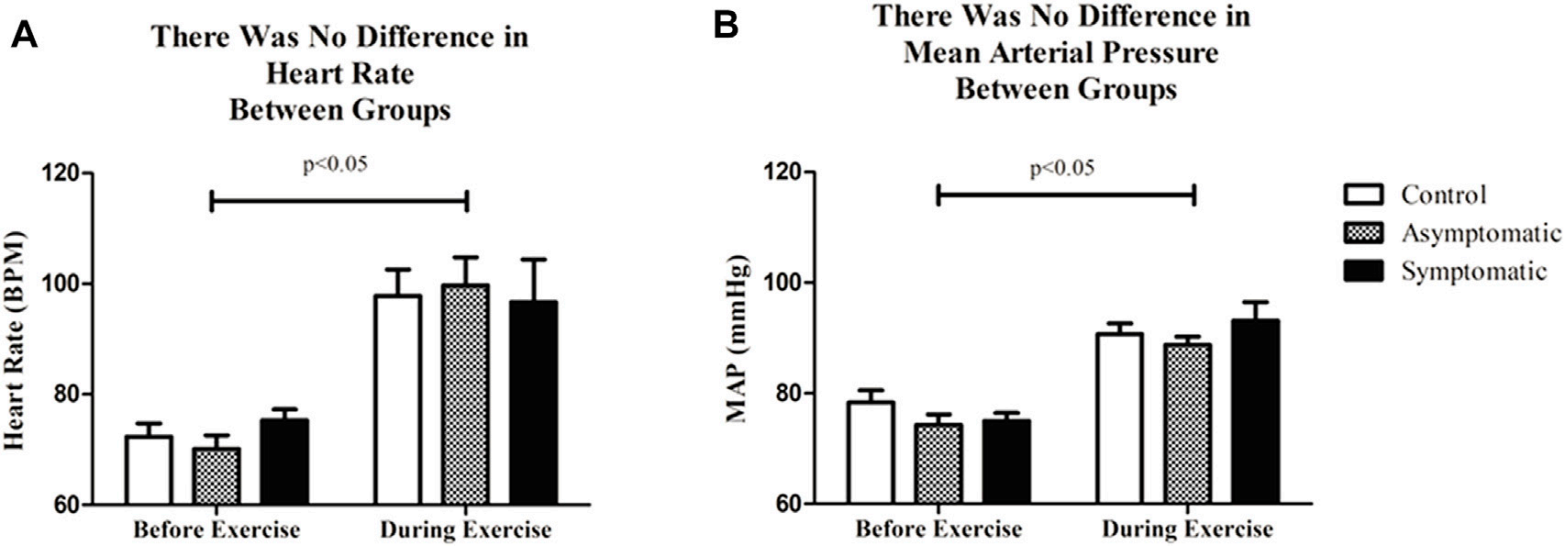
**(A)** There was no difference in average heart rate or **(B)** MAP before or during submaximal treadmill exercise between groups. *n* = 9/group; Data analyzed by a Two-Way Repeated Measures ANOVA with a Bonferroni post-hoc. Data are presented as mean ± SE; HR = heart rate; MAP = mean arterial pressure. Significance was set to *p* < 0.05.

**FIGURE 3:**
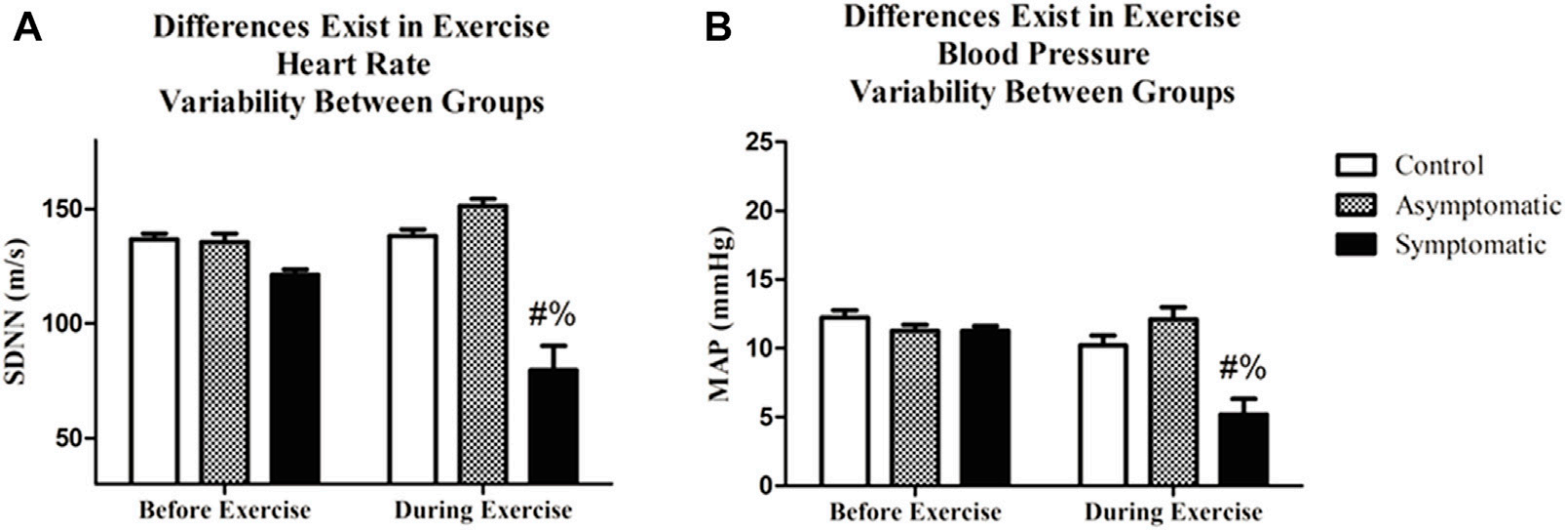
Exercise significantly altered both heart rate variability (HRV) and blood pressure variability (BPV). Additionally, **(A)** HRV was lower during exercise in COVID-19 symptomatic individuals relative to asymptomatic and control. **(B)** BPV was different in the symptomatic group during exercise relative to the asymptomatic and control groups. Data analyzed by by a Two-Way Repeated Measures ANOVA with a Bonferroni post-hoc. # = symptomatic vs. control; and = symptomatic vs asymptomatic. Data are presented as mean ± SE; HRV = heart rate variability; BPV = blood pressure variability. Significance was set to *p* < 0.05.

Following resting blood pressure, we initiated submaximal exercise. The average heart rate and blood pressure throughout the exercise bout, which excluded warm-up and cooldown, are presented in Figure 2. Not surprisingly there was a main effect of exercise for both heart rate and MAP. We noted no differences amongst groups. However, when we examined HRV and BPV in the time-domain both variables (Figure 3) were significantly different in the S group during exercise. There was a main effect for the group (Df = 2; F = 29.35) and the S group had a reduction in (*p* < 0.05) SDNN (S = 79.6 ± 10.7 m) relative to both AS and the control subjects (Control = 138.2 ± 2.8 m; AS = 151.4 ± 3.1 m). We noticed the same trend for BPV; the S group (S = 5.2 ± 1.5 mmHg) had a reduction in variability (*p* < 0.05) when compared with AS and our controls (CON = 10.2 ± 0.73 mmHg AS = 12.1 ± 0.88 mmHg).

**FIGURE 4:**
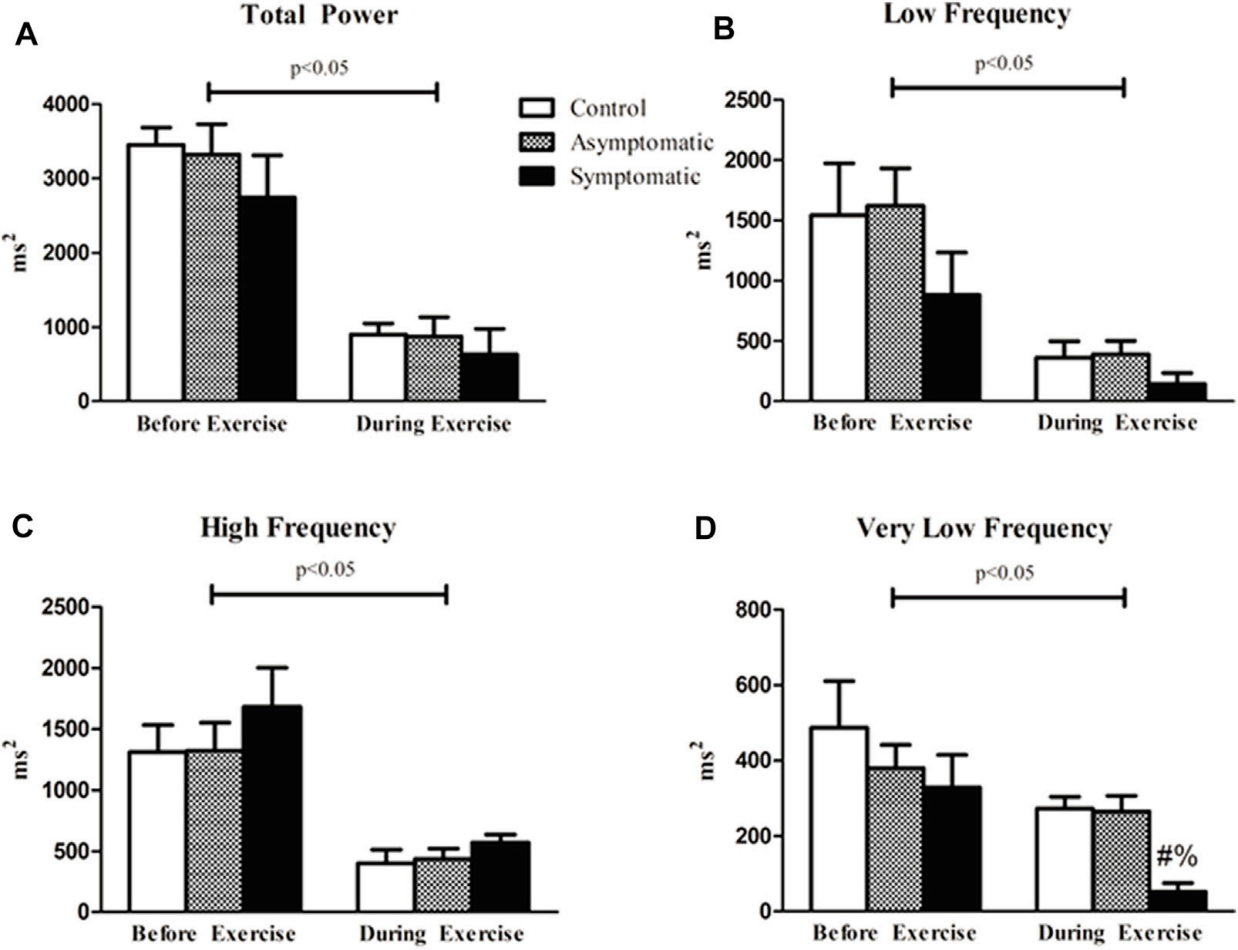
Spectral analysis of HRV revealed that there was an effect of exercise in all four variables. However, there was no difference between groups for **(A)** total power (TP) both before and after exercise. **(B)** High frequency (HF) and **(C)** low frequency (LF) bands were not different between our groups. **(D)** Very low frequency (VLF) power analysis revealed that the symptomatic (S) group had lower power relative to the other groups during exercise. Data analyzed by a Two-Way Repeated Measures ANOVA with a Bonferroni post-hoc. # = symptomatic vs. control; and = symptomatic vs. asymptomatic; Data are presented as mean ± SE; HRV = heart rate variability; BPV = blood pressure variability. Significance was set to *p* < 0.05.

We next performed spectral analysis on our heart rate data. Data is represented in Figure 4. Our results our represented by each frequency domain both before and after exercise. There was a main effect of exercise for all four variables but no differences were found for the HF and LF bands. However, the VLF band was significantly reduced and there was a main effect for group (Df = 2; F = 3.824). There was no difference before exercise in our S group but there was a reduction VLF power during exercise (CON = 273 ± 31 m^2^; AS = 265 ± 42 m^2^; S = 52 ± 23 m^2^). When we assessed our data for violations of stationary we determined that the majority of our participants were non-stationery (*p* > 0.05) and failed to reject the null-hypothesis. Our control subjects appeared to have the most stable signal with 58% stationary throughout exercise while 42% and 37% of our AS and S participants were stationary, respectively.

**FIGURE 5:**
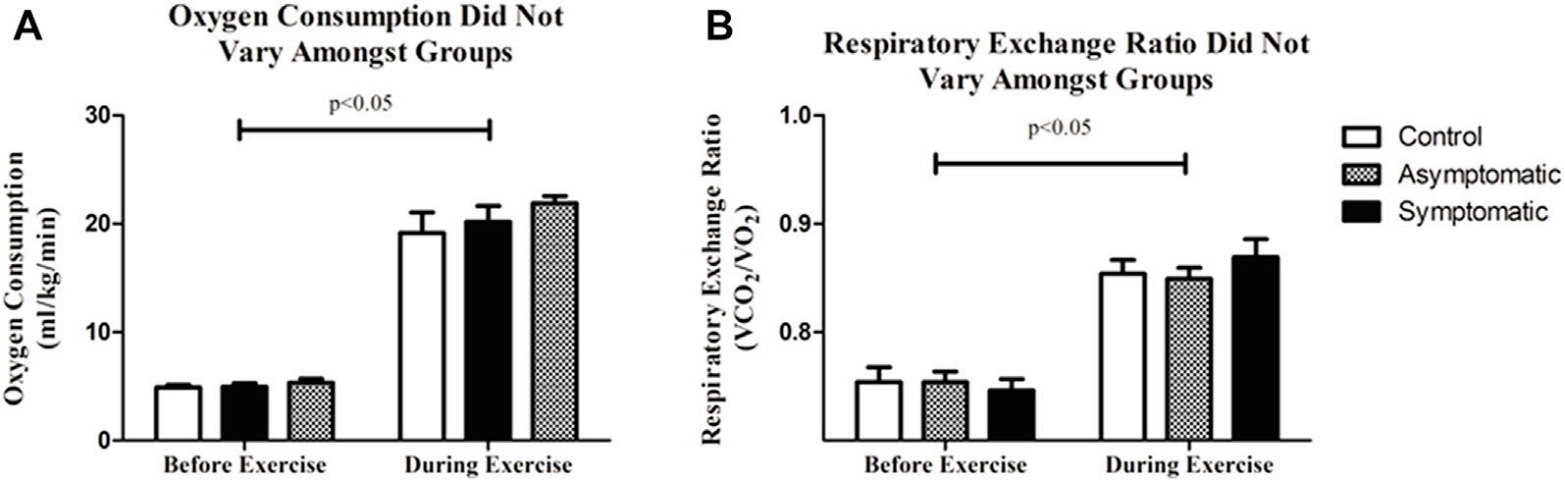
**(A)** There was no difference in oxygen consumption (VO_2_) between all three groups. **(B)** Respiratory exchange ratio (RER) was consistent amongst groups. Data analyzed by a Two-Way Repeated Measures ANOVA with a Bonferroni post-hoc. Data are presented as mean ± SE; VO_2_ = oxygen consumption; RER = respiratory exchange ratio.

We examined VO_2_ and respiratory exchange ratio (RER) to look for potential difference in respiratory function. We noticed no differences amongst our groups. VO_2_ was consistent between all three groups (CON = 21.7 + 0.6 mL/kg/min; AS = 20.5 + 1.5 mL/kg/min; S = 21.9 + 0.7 mL/kg/min) as was RER and increased with exercise (Figure 5). Although, some previous reports demonstrated an increase in carbon dioxide expiration (RER) in previously COVID-19 positive patients (Singh et al., 2022); we noticed no difference in our subjects throughout this particular bout of exercise.

## Discussion

Medical researchers now believe that SARS-CoV-2 has the ability to cause acute changes to the autonomic nervous system as well as cause other chronic cardiovascular and neurological conditions (Lotfi et al., 2020; Wu et al., 2020; Veluswamy et al., 2021). We also need to identify if differences exist between COVID-19 infected individuals, who had symptoms (symptomatic) or did not have symptoms (asymptomatic) is important. Our laboratory’s data suggests an altered HRV and BPV response to submaximal exercise in previously symptomatic and asymptomatic COVID-19 positive individuals and those who never contracted SARS-CoV-2.

Due to the increase in cellular oxygen demand, which is attributed to exercise, heart rate must increase in a near linear relationship which is necessary to increase and maintain exercise intensity. Furthermore, because blood pressure is relative to cardiac output, arterial pressure follows the same relationship for the duration of exercise. Adjustments are made second by second though pressure and chemical sensitive receptors to ensure oxygen supply and hydrogen removal are adequate (Savytskyi et al., 2022). Therefore, it has been very well established that the autonomic nervous system is vital for completion of both short- and long-term exercise bouts (Savytskyi et al., 2022).

Altered HRV and BPV in general can indicate potential issues with one’s health (Tsuji et al., 1996; Routledge et al., 2010). Previous studies have shown that reduced HRV is an indication of poor cardiovascular health and can be used as a potential risk factor for stroke (Fyfe-Johnson et al., 2016). Chronic exercise has previously been shown to normalize HRV following myocardial ischemia in both dogs and humans and drastically increased survival rates (Grässler et al., 2021). BPV is another marker of cardiovascular health and during cardiovascular exercise sessions a decrease in blood pressure control is demonstrated in several disease states including obesity (Dipla et al., 2012) and type II diabetes (Grotle et al., 2012). Thus, there is an apparent link between HRV and BPV with exercise and general cardiovascular health. This can be of obvious concern for individuals who had prior bout of symptomatic COVID-19; particularly those with other underlying risk factors (obesity, hypertension, etc*.*).

SDNN can be a potential indicator of both sympathetic and parasympathetic activity (Shaffer and Ginsberg, 2017). However, during short-term recordings parasympathetic activity is likely the cause for most variation in SDNN (Shaffer et al., 2014; Shaffer and Ginsberg, 2017). Time-domain measurements may not be enough to fully quantify autonomic control (La Rovere et al., 2020), we therefore examined HRV in the frequency domain. SDNN appears to correlate with low frequency rhythms (Umetani et al., 1998; Shaffer and Ginsberg, 2017). Indeed, we found alterations in the VLF band of the COVID-19 sympathetic group relative to our other groups. Although there is some question of the physiological mechanisms of the VLF band it has been shown to be strongly associated to arrhythmic death and low power in this band is significantly related to markers of inflammation (Carney et al., 2007; Shaffer et al., 2014). This is in line with past studies showing that past SARS-CoV2 infections can alter cardiac function and lead to an extended inflammatory response (Mascia et al., 2021; Mermutluoğlu et al., 2022). There is also evidence which suggests that VLF is modulated by parasympathetic activity as parasympathetic blockade abolished VLF power while sympathetic blockade had no effect (Berntson et al., 1997; Shaffer et al., 2014). Lastly, we need to caution against a potential overestimation of the contribution of the parasympathetic nervous system to the variances seen in our data. The occurrence of non-stationaries in our data set can lead to a bias in our data set (Magagnin et al., 2011).

We also saw a decrease in BPV during exercise in our S group. While high BPV correlates with pulse wave velocity and vascular remodeling (Parati et al., 2018). Low BPV may an indicator of a decrease in cardiopulmonary reflex (Parati et al., 2018). Being that the cardiopulmonary reflex responds to an increase in central pressure and changes in human chemical state it is crucial for the regulation of blood pressure during exercise. However, it is quite difficult to make this conclusion based on the data from this study. Further, a low BPV can also be indicative of behavioral and emotional status making it hard to make strong assumptions (Parati et al., 2018).

It has been already reported that exercise intolerance is a long-term effect of a prior COVID-19 infection (Ferreira and Oliveira, 2021). Further, these studies have demonstrated an aerobic detraining effect, an increase in carbon dioxide expiration and endothelial dysfunction (Rinaldo et al., 2021; Skjørten et al., 2021). These issues can be exacerbated even further by conditions that often go undetected such as hypertrophic cardiomyopathy, which can contribute to sports-associated sudden death (Mascia et al., 2021). Along these same lines genetic test and echocardiography performed early in an athlete’s career can lead to improvements the management of genetic conditions such right ventricular arrhythmogenic cardiomyopathy (Odak et al., 2022). Collectively, our data along with previous data demonstrating potential changes to athlete’s hearts who were COVID-19 positive (de Abreu, 2022) suggests the need for improvements in the medical screening process for individuals prior to the initiation exercise or sport. Specifically, this study provides some evidence that previously symptomatic COVID-19 patients have an altered HR and BP response. While our subjects were all able to finish a moderate intensity bout of exercise it is important to consider the fact that this population were all young and otherwise healthy individuals. Further, our results could have varied if our subjects were taken to high intensity or maximal exercise.

In conclusion our data suggests an altered blood pressure and heart response to submaximal exercise in individuals who previously displayed symptoms from COVID-19 when compared to those who contracted the virus but remained asymptomatic. Specifically, these individuals may have instances of reduced BPV and HRV during submaximal exercise. Further, a decrease in SDNN and the VLF band may suggests an alteration in parasympathetic function during exercise. This may be a contributing factor to the previously documented COVID-19 related exercise intolerance. Future studies should build off of these data and combine similar types of analysis with biochemical assessments.

We acknowledge that our study has several limitations. Our subject pool was undersized and limited to college-aged students. Much of our data was collected during a partial lockdown period and therefore we were unable to recruit as freely as we wished. Also, a larger sample size would have allowed United States more robust statistical analysis. Some significant findings may have been masked due to low numbers. Secondly, another important limitation was we possessed no biochemical evidence that there was indeed a prior infection. Cardiac Troponin I has the potential to be an independent predictor of SARS-CoV2 related mortality (Mascia et al., 2021). This is particularly true in severe cases where myocarditis may be present. Further, increased ferritin levels and decreased lymphocyte count appear to correlate with the length of hospital stay in COVID-19 patients (Mermutluoğlu et al., 2022). Finally, we acknowledge that spectral analysis of systolic and diastolic blood pressure is more suitable for describing sympathetic control than the methods employed here and therefore our BPV data is harder to interpret (Bosso et al., 2020).

The corresponding author has received funding from Bristol Myers Squib. There are no further financial or other relationships that could lead to a conflict of interest with this manuscript. All authors have declared that no conflict of interest exists.

## Data Availability

The raw data supporting the conclusions of this article will be made available by the authors, without undue reservation.
